# Effects of One Resistance Exercise Session on Vascular Smooth Muscle of Hypertensive Rats

**DOI:** 10.5935/abc.20150070

**Published:** 2015-08

**Authors:** Tharciano Luiz Teixeira Braga da Silva, Marcelo Mendonça Mota, Milene Tavares Fontes, João Eliakim dos Santos Araújo, Vitor Oliveira Carvalho, Leonardo Rigoldi Bonjardim, Márcio Roberto Viana Santos

**Affiliations:** Universidade Federal de Sergipe; Universidade de São Paulo – Brazil

**Keywords:** Hypertension, Exercise, Vasodilatation, Rats, Muscle, Smooth, Mesenteric, Artery

## Abstract

**Background:**

Hypertension is a public health problem and increases the incidence of cardiovascular diseases.

**Objective:**

To evaluate the effects of a resistance exercise session on the contractile and relaxing mechanisms of vascular smooth muscle in mesenteric arteries of N^G^-nitro L-arginine methyl ester (L-NAME)-induced hypertensive rats.

**Methods:**

Wistar rats were divided into three groups: control (C), hypertensive (H), and exercised hypertensive (EH). Hypertension was induced by administration of 20 mg/kg of L-NAME for 7 days prior to experimental protocols. The resistance exercise protocol consisted of 10 sets of 10 repetitions and intensity of 40% of one repetition maximum. The reactivity of vascular smooth muscle was evaluated by concentration‑response curves to phenylephrine (PHEN), potassium chloride (KCl) and sodium nitroprusside (SNP).

**Results:**

Rats treated with L-NAME showed an increase (p < 0.001) in systolic blood pressure (SBP), diastolic blood pressure (DBP) and mean arterial pressure (MAP) compared to the initial period of induction. No difference in PHEN sensitivity was observed between groups H and EH. Acute resistance exercise reduced (p < 0.001) the contractile response induced by KCl at concentrations of 40 and 60 mM in group EH. Greater (p < 0.01) smooth muscle sensitivity to NPS was observed in group EH as compared to group H.

**Conclusion:**

One resistance exercise session reduces the contractile response induced by KCl in addition to increasing the sensitivity of smooth muscle to NO in mesenteric arteries of hypertensive rats.

## Introduction

Hypertension is a public health problem worldwide, and is associated with the increasing incidence of deaths due to cardiovascular diseases^1^. Several hypertension models have been developed within the basic sciences to mimic the pathological effects of hypertension^2,3^. The experimental hypertension model in rats using inhibition of nitric oxide synthase (NOS) with N^G^-nitro-L-arginine-methyl-ester (L-NAME) determines arterial hypertension, kidney injury, sympathetic overactivity and endothelial dysfunction^4-8^.

It is worth noting that the induction of hypertensive rats depends on the L-NAME dose administered, treatment duration, target organ studied, age and type of the animal used in the study. In association with that hypertension model, studies have shown that aerobic and resistance exercises are beneficial regarding aspects related to blood pressure and vascular function in rats^9,10^.

Nitric oxide synthase inhibition induces hypertension by increasing blood pressure via an endothelium-dependent response^7^. Our team has recently shown that submitting L-NAME-induced rats to resistance exercise for four weeks can reduce sensitivity to phenylephrine (PHEN) and increase sensitivity to sodium nitroprusside (SNP) of the superior mesenteric artery smooth muscle^10^.

The study of resistance exercise in animal models mimicking hypertension provides relevant information for clinical studies aimed at disease prevention, treatment and control. Despite our team’s findings^10^, so far the effects of resistance exercise on the contracting and relaxing parameters related to vascular smooth muscle have not been well established. A study has recently shown that submitting spontaneously hypertensive rats (SHR) to one session of resistance exercise does not change the vascular function of the tail artery in relaxations induced by SNP, an exogenous donor of nitric oxide (NO)^11^. Several variables, such as disease animal model, type of artery studied, type of resistance exercise, and volume, intensity and duration of physical stimulus, can influence the benefits of resistance exercise. The present study aimed at assessing the effects of one resistance exercise session on the contraction and relaxation mechanisms of the mesenteric artery smooth muscle of L-NAME-induced hypertensive rats.

## Methods

### Animals

Wistar rats (250-300 g) were used in all experiments. The animals were maintained under controlled temperature (22 ± 1°C) and 12-hour light-dark cycles, with water and food *ad libitum*. All procedures described in the present study were approved by the Ethics Committee in Research with Animals of the Universidade Federal de Sergipe, Brazil (Protocol 32/2013). The animals were divided into three groups with ten animals each: sedentary control (C); sedentary control with hypertension (H) and exercised hypertensive (EH). The animals of groups C and H were maintained inside boxes with no exposure to exercise, and only group EH animals underwent one resistance exercise session.

### Hypertension induction, and blood pressure and body weight measurements

Before beginning the procedure of experimental hypertension induction, blood pressure was measured by using the non-invasive caudal method (LETICA, LE5002, Barcelona, Spain). After that, only animals of groups H and EH received orally L-NAME (20 mg/kg, daily), through gavage, for seven days^8^. By the end of the induction period, blood pressure was measured again in all groups. Animals with mean arterial pressure (MAP) greater than 130 mm Hg were categorized as hypertensive. Body weight was daily assessed to adjust the L-NAME dosage.

### Protocol of resistance exercise

Resistance exercise was performed in a squatting apparatus according to the model by Tamaki et al.^12^. Initially, group EH animals were acquainted with the exercise apparatus for three days, and, then, the one repetition maximum (RM) test was performed. One RM was determined as the maximum weight lifted by each rat, using the exercise apparatus^13^. Two days after the RM test, the animals underwent the resistance exercise protocol adapted from Fontes et al.^14^. The rats underwent ten sessions of ten repetitions, with 60-second rest intervals, and intensity of 40% of the load established by using the RM test. The parameters of electrical stimulation are similar to those described by Barauna et al.^15^. The animals of groups C and H underwent none of those procedures.

### Assessing smooth muscle vascular reactivity

Immediately after the resistance exercise session, all rats of all groups were sacrificed and superior mesenteric artery rings, free from connective tissue, were sectioned (1-2 mm). Endothelium-independent relaxation was assessed by using the superior mesenteric artery rings prepared according to the description by Menezes et al.^16^. The presence or absence of functional endothelium was assessed by the ability, measured as percentage (%), of acetylcholine (ACh; 1 µM) to relax the pre-contracted rings with 1 µM of PHEN. Rings whose relaxations were below 10% were considered not to have a functional endothelium and automatically selected for this study^17^.

The changes in vascular reactivity due to the contracting and relaxing agents were assessed through concentration-response curves of the superior mesenteric artery rings of the rats of all groups. After the stabilization period of the isolated rings, curves for the contracting agents were performed: PHEN (10^-9^-10^-4^ M) (α-1 adrenergic agonist) and KCl (20-80 mM) (unspecific contracting agent). In addition, experiments for the relaxing agent were conducted: SNP (10^-11^-10^-6^ M), NO donor, in pre-contracted rings with PHEN (1 µM). All experimental protocols were conducted separately.

Data from the concentration-response curves were assessed by using the adjustment of a logistic function: E = Rmax/((1+(10c/10x)n) + Φ), where E is the response; Rmax is the maximal response the agonist can produce; c is the logarithm of EC_50_, which is the concentration at which the agonist produces a response equal to 50% of the maximal response; x is the logarithm of the concentration of the agonist; the exponential term, n, is a parameter of adjustment of the curve that defines the inclination of the concentration-response line; and Φ is the response observed in the absence of the agonist. Non-linear regression analyses were performed to determine the parameters Rmax, EC_50_ and n, with the restriction Φ = zero. The sensitivity of the superior mesenteric artery rings was assessed by determining the pD_2_ value of each agonist. That corresponds to the negative logarithm of the molar concentration of the agonist that determines a response equal to 50% of the maximal response (EC_50_), in each experiment.

### Drugs and reagents

N^G^-nitro-L-arginine-methyl-ester (L-NAME), acetylcholine chloride (ACh), L-phenylephrine chloride (PHEN), sodium nitroprusside (SNP), salts and reagents used in the present study were obtained from Sigma (Sigma Chemical Co, St. Louis, MO, USA).

### Statistical analyses

The Kolmogorov-Smirnov test was used to determine whether the probability distributions of the data were parametric or non-parametric. All data had a normal distribution. The values were expressed as mean ± standard error of the mean (SEM). Student *t* tests paired and analysis of variance (one-way and two-way ANOVA) followed by Bonferroni post-test were used when necessary to assess the significance of the differences between the means. The values were considered statistically significant when p < 0.05. The GraphPad Prism program, version 3.02 (GraphPad Software, San Diego-CA, USA), was used in all procedures.

## Results

### Body weight and blood pressure in response to hypertension induction

We observed that in the beginning and end of the hypertension induction period, the body weight of the rats was similar in all groups. After seven days of induction, the rats treated with L-NAME showed an increase (p < 0.001) in the levels of MAP, systolic blood pressure (SBP) and diastolic blood pressure (DBP). When the end of the induction period between the groups was statistically assessed, L-NAME showed to induce an increase in MAP, SBP and DBP (p < 0.001) in the groups H and EH as compared to group C (Table 1).

**Table 1:** Body weight, mean arterial pressure (MAP), systolic blood pressure (SBP) and diastolic blood pressure (DBP) of rats at the beginning and end of systemic hypertension induction

GROUPS	PERIOD	WEIGHT (g)	MAP (mm Hg)	SBP (mm Hg)	DBP (mm Hg)
C (n = 10)	INITIAL	253 ± 12.0	101.6 ± 1.8	125.0 ± 1.6	90.0 ± 2.0
	FINAL	258 ± 13.7	106.3 ± 2.1	129.0 ± 1.4	95.0 ± 2.2
H (n = 10)	INITIAL	257 ± 11.6	104.3 ± 1.4	121.0 ± 1.5	96.0 ± 2.1
	FINAL	263 ± 13.6	134.3 ± 2.0***.^C^	147.0 ± 1.8***.^C^	128.0 ± 1.9***.^C^
EH (n = 10)	INITIAL	252 ± 12.6	104.6 ± 1.7	128.0 ± 1.3	93.0 ± 2.3
	FINAL	257 ± 14.6	131.9 ± 1.9***.^C^	145.0 ± 1.3***.^C^	124.0 ± 1.6***.^C^

C: control group; H: hypertensive group; EH: exercised hypertensive group. Data are shown as means ± SEM. The statistical differences were determined by Student t tests one-way ANOVA followed by Bonferroni post-test.

*** p < 0.001 initial vs final period;

C p < 0.001 vs final period of the control group.

### Vascular smooth muscle constriction in response to PHEN

**Figure 1:**
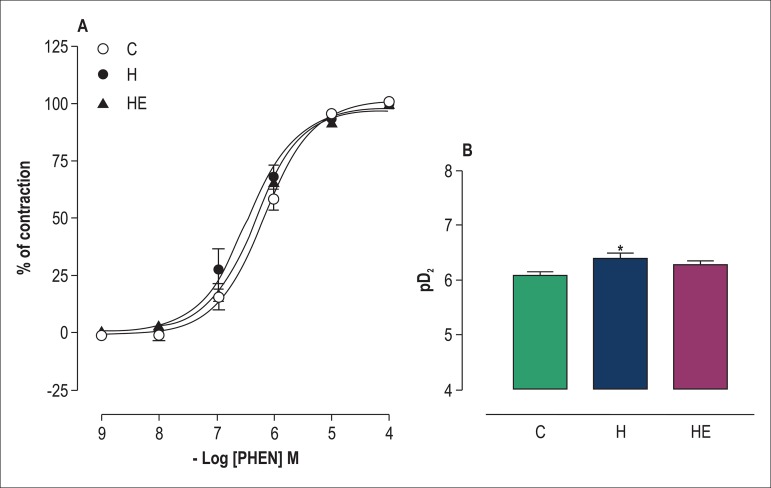
Concentration-response curves for phenylephrine (PHEN: 10^-9^-10^-4^ M) in isolated superior mesenteric artery rings without functional endothelium (Figure 1A) obtained from rats of the groups Control (C), Hypertensive (H) and Exercised Hypertensive (EH). Figure 1B indicates means ± standard error of the mean (SEM) of pD_2_ of the phenylephrine induced contractions (B). Data are expressed as means ± SEM for ten experiments in each group. The statistical differences between means were determined by using two-way ANOVA followed by the Bonferroni post-test (Figure 1A) and one-way ANOVA followed by the Bonferroni post-test (Figure 1B). *p < 0.01 vs C. pD_2_: negative logarithm of the molar concentration of the agonist that produces 50% of maximal response.

We observed that PHEN (10^-9^-10^-4^ M) induced concentration-dependent contraction of the isolated rings of the superior mesenteric artery in all groups. However, the maximal contraction response did not differ between the groups (Figure 1A).

L-NAME could interfere with the arterial sensitivity of PHEN-induced contractions in hypertensive-induced rats, because pD_2_ changed (p < 0.05) in group H as compared to group C (Figure 1B). In addition, one resistance exercise session did not interfere with arterial sensitivity, and pD_2_ remained unaltered when comparing groups EH and H (Figure 1B).

### Vascular smooth muscle constriction in response to KCl

**Figure 2:**
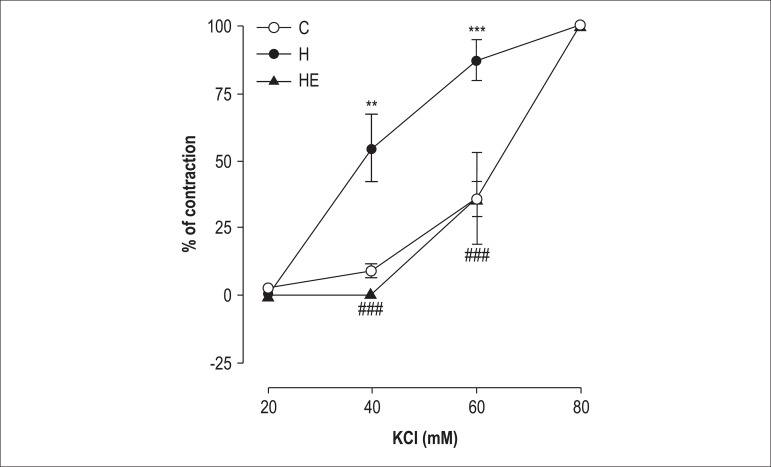
Concentration-response curves for potassium chloride (KCl: 20-80 mM) in isolated superior mesenteric artery rings without functional endothelium obtained from rats of the groups Control (C), Hypertensive (H) and Exercised Hypertensive (EH). Data are expressed as means ± standard error of the mean (SEM) for ten experiments in each group. The statistical differences between means were determined by using two-way ANOVA followed by the Bonferroni post-test. **p < 0.01 and ***p < 0.001 vs C; ^###^p < 0.01 and ^###^p < 0.001 vs H.

We observed that the increase in extracellular KCl (20-80 mM) produced contractile tension in the isolated rings of the superior mesenteric artery of the rats of all groups. However, the maximal responses induced by KCl did not differ between the groups (Figure 2). The animals induced to hypertension with L-NAME had a higher percentage contraction of vascular smooth muscle at the concentrations of 40 and 60 mM of KCl (p < 0.01; p < 0.001, respectively) (Figure 2). On the other hand, group EH animals had a lower percentage contraction of vascular smooth muscle at the concentrations of 40 and 60 mM (p < 0.001) (Figure 2).

### Vascular smooth muscle dilation in response to SNP

**Figure 3:**
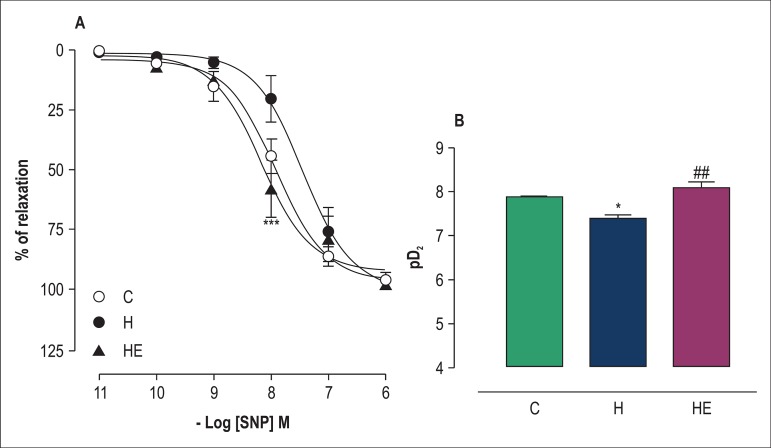
Concentration-response curves for sodium nitroprusside (SNP: 10^-11^-10^-6^ M) in isolated superior mesenteric artery rings without functional endothelium and pre-contracted with phenylephrine (1 µM) (Figure 3A). The rings were obtained from rats of the groups Control (C), Hypertensive (H) and Exercised Hypertensive (EH). Figure 3B indicates means ± standard error of the mean (SEM) of pD_2_ of the SNP-induced relaxations. Data are expressed as means ± SEM for ten experiments in each group. The statistical differences between means were determined by using two-way ANOVA followed by the Bonferroni post-test (Figure 3A) and one-way ANOVA followed by the Bonferroni post-test (Figure 1B). *p < 0.05 vs C; ***p < 0.001 vs H; ^##^p < 0.01 vs H. pD_2_: negative logarithm of the molar concentration of the agonist that produces 50% of maximal response

We observed that SNP (10^-11^-10^-6^ M) induced endothelium-independent relaxation in the isolated rings of the superior mesenteric artery of the rats of all groups (Figure 3A). The maximal vascular relaxation in response to SNP was similar in the three groups studied (Figure 3A). L-NAME reduced (p < 0.05) the arterial sensitivity to SNP in group H animals as compared to those in group C (Figure 3B).

Inversely, we observed that resistance exercise could restore arterial sensitivity to SNP by increasing (p < 0.01) pD_2_ of group EH as compared to that of group H (Figure 3B).

## Discussion

The results of this study show that one resistance exercise session in L-NAME-induced hypertensive rats caused a reduction in the KCl-induced contracting mechanisms by increasing the vasodilating sensitivity of the mesenteric artery smooth muscle.

There is evidence that the reduced levels of NO play an important role in the development of hypertension^4,18^. The experimental model of hypertension that mimics that effect is the one induced by the inhibition of NOS with a unspecific inhibitor, L-NAME^4,19^. Treatment with L-NAME is associated with structural and functional changes in the kidneys, changes in autonomic modulation and in peripheral vascular resistance, and an increase in blood pressure^4,6,8,20^. The present study showed a blood pressure increase of the animals treated with L-NAME for seven days. The hypertensive levels obtained are similar to those previously reported for rats treated with L-NAME for seven days^6,8,20^.

The literature describes that the transmission of the signal originated in the plasma membrane for the receptors of the smooth muscle contractile machinery is due to pharmacomechanical and/or electromechanical stimuli^21^. Those mechanisms should not be understood as completely separated systems, but understood as part of a network of signals that interact to maintain vascular physiology. In our study, the rats treated with L-NAME showed higher α-1 adrenergic sensitivity. The modulation of α-1 adrenergic receptors and the reduction in NO production play an important role in the cardiovascular changes of hypertensive rats^22^. It has already been shown that a reduction in NO shifts the contraction curve of PHEN to the left in the aorta of rats, but not in the tail artery, confirming that the modulation of receptors in response to NO seems to depend on the type of the artery studied^23^. Heijenbrok et al.^24^ have treated *Wistar* rats with L-NAME (15 mg/kg/day) for six weeks and have not observed any change related to PHEN in the vascular reactivity of the carotid and mesenteric arteries. The inconsistence about the modulation of α-1 adrenergic receptors in animals treated with L-NAME can be associated with the administration route, the drug dose, the treatment length and the type of artery studied.

In our study, immediately after one low-intensity resistance exercise session, there was no change in the sensitivity of α-1 adrenergic receptors of rats treated with L-NAME. In healthy rats, repeated strenuous swimming sessions, rather than only one session, reduced α-1 adrenergic sensitivity in the mesenteric artery with injured endothelium^25^. Our results differ from those by Faria et al.^11^, who, after one resistance exercise session (20 x 15, 50% intensity), have shown greater attenuation of the post-exercise responses to PHEN in the tail artery with intact endothelium^11^. The differences in our results can be attributed to the training protocol, the experimental hypertension model, the type of artery studied and the functional endothelium preservation to assess vascular reactivity. On the other hand, a previous study of our group has demonstrated that chronic low-intensity resistance exercise (3 x 10, 50% intensity) controlled blood pressure and reduced the α-1 adrenergic sensitivity of the mesenteric artery without functional endothelium of L-NAME-induced hypertensive rats^10^. This shows that one low-intensity resistance exercise session in L-NAME-induced hypertensive rats does not seem to be sufficient to make a change at the α-1 adrenergic receptor level, but successive resistance exercise sessions can cause a significant reduction in the contractile sensitivity promoted by PHEN.

In addition, the present study assessed another mechanism that modulates smooth muscle contraction, the contractile coupling through depolarizing KCl solutions. In general, KCl produces smooth muscle vascular contraction via membrane depolarization, causing Ca^2+^ inflow via voltage-dependent Ca^2+^ channels^26^. It has been reported that depolarizing KCl concentrations mediate the increase in intracellular Ca^2+^ concentration^27^. Our results indicate that animals treated with L-NAME increased smooth muscle contraction through membrane depolarization in mesenteric artery rings. Other studies with chronically L-NAME-induced animals have shown abnormal functioning of the voltage-dependent Ca^2+^ channels^5,28^. Bank et al.^28^ have suggested that the L-NAME-induced hypertension model increases the vascular smooth muscle tonus, and such effect is due to the reduction in NOS availability, which can lead to an increase in Ca^2+^ concentration or intracellular sensitivity. These findings are in accordance with our results that the increase in smooth muscle contractility found in L-NAME-induced animals can be related to the KCl-induced contractile mechanisms.

It is worth noting that, when the rats in our study underwent one resistance exercise session, they had a reduction in contraction in response to depolarizing KCl solutions (20-80 mM). That points to the possibility that resistance exercise alters in a beneficial way the depolarization of the vascular smooth muscle cells of L-NAME-induced hypertensive animals. Similarly, Chen et al.^29^ have shown a reduction in the contractile response to KCl (15-60 mM) in mesenteric artery rings of healthy rats after eight weeks of running training. In addition, the aortic rings of rats trained in running (10 to 12 weeks) have shown a lower contractile response to depolarizing KCl concentrations (10-100 mM) by the end of the protocol^30^. So far, the effects of resistance exercise on the contractile response of the smooth muscle to depolarizing KCl solutions have not been described. The present study is the first to show the efficacy of one resistance exercise session on the decrease of smooth muscle contractility via independent mechanisms of adrenergic receptors in hypertensive rats. These results suggest that low-intensity resistance exercise, when performed for a long period, can be an important tool to fight cardiovascular disorders originating from smooth muscle contractile mechanisms.

In addition, we observed that the rats treated with L-NAME had lower vasodilating sensitivity to SNP. When submitted to one resistance exercise session, they showed increased vasodilating sensitivity to NO in the smooth muscle of mesenteric artery rings. A recent study by our group has shown that the NO pathway sensitivity was decreased in L-NAME-induced hypertensive rats for eight weeks, and that chronic low-intensity resistance exercise could reverse that effect^10^. Acute and chronic effects of resistance exercise on the endothelium-independent vasodilating response are beneficial to vascular function in L-NAME-induced hypertensive rats. It is worth noting that, the study by another group conducting one resistance exercise session in spontaneously hypertensive animals has shown no changes in the SNP-induced relaxations in the vascular bed of the tail artery^25^. Those differences can result from the hypertension induction model and the training protocol adopted.

The present study has some limitations. The first is that the results obtained are specific to hypertensive rats. The second is not having assessed the effect of resistance exercise on other arteries because there already is functional heterogeneity among the arteries of different vascular beds. The third is the lack of a healthy group undergoing exercise, which limits data extrapolation. Another point to be noted is that the resistance exercise protocol adopted in the present study has the characteristic of high volume and low intensity. That exercise characteristic is similar to aerobic exercise protocols indicated to control blood pressure^1,13^. Some studies have shown that moderate-intensity resistance exercise can reduce blood pressure and improve vascular function^1,11,31,32^. Despite those advantages, a recent meta-analysis has indicated that high-intensity resistance exercise is associated with increased arterial stiffness in health young individuals^33^. In addition, the physiological mechanisms responsible for the advantages and/or disadvantages of resistance exercise on the vascular health of animals and humans are yet to be established.

## Conclusion

The pharmacological evidence of this study showed that one resistance exercise session caused benefits to the vascular function of L-NAME-induced hypertensive animals. Those benefits involve a reduction in the contractile responses via KCl-induced cell depolarization, independent of α-1 adrenergic receptors, and higher vasodilating sensitivity to NO of the mesenteric artery smooth muscle in L-NAME-induced hypertensive rats. The vascular smooth muscle adjustments resulting from one resistance exercise session seem beneficial to control vascular tonus in hypertension.

## References

[1] Pescatello LS, Franklin BA, Fagard R, Farquhar WB, Kelley GA, Ray CA, American College of Sports Medicine (2004). American College of Sports Medicine position stand. Exercise and
hypertension. Med Sci Sports Exerc.

[2] Török J (2008). Participation of nitric oxide in different models of experimental
hypertension. Physiol Res.

[3] Dornas WC, Silva ME (2011). Animal models for the study of arterial
hypertension. J Biosci.

[4] Ribeiro MO, Antunes E, de Nucci G, Lovisolo SM, Zatz R (1992). Chronic inhibition of nitric oxide synthesis. A new model of
arterial hypertension. Hypertension.

[5] Ribeiro MO, Antunes E, Muscará MN, De Nucci G, Zatz R (1995). Nifedipine prevents renal injury in rats with chronic nitric
oxide inhibition. Hypertension.

[6] Souza HC, Ballejo G, Salgado MC, Da Silva VJ, Salgado HC (2001). Cardiac sympathetic overactivity and decreased baroreflex
sensitivity in L-NAME hypertensive rats. Am J Physiol Heart Circ Physiol.

[7] Török J, Kristek F (2001). Functional and morphological pattern of vascular responses in two
models of experimental hypertension. Exp Clin Cardiol.

[8] Biancardi VC, Bergamaschi CT, Lopes OU, Campos RR (2007). Sympathetic activation in rats with L-NAME-induced
hypertension. Braz J Med Biol Res.

[9] Kuru O, Sentürk UK, Koçer G, Ozdem S, Baskurt OK, Cetin A (2009). Effect of exercise training on resistance arteries in rats with
chronic NOS inhibition. J Appl Physiol (1985).

[10] Araujo AJ, Santos AC, Souza KS, Aires MB, Santana VJ, Fioretto ET (2013). Resistance training controls arterial blood pressure from L-NAME
induced hypertensive rats. Arq Bras Cardiol.

[11] Faria T de O, Targueta GP, Angeli JK, Almeida EA, Stefanon I, Vassallo DV (2010). Acute resistance exercise reduces blood pressure and vascular
reactivity, and increases endothelium-dependent relaxation in spontaneously
hypertensive rats. Eur J Appl Physiol.

[12] Tamaki T, Uchiyama S, Nakano S (1992). A weight-lifting exercise model for inducing hypertrophy in the
hindlimb muscles of rats. Med Sci Sports Exerc.

[13] Pescatello LS, Arena R, Riebe DW, Thompson PD (2013). ACSM's guidelines for exercise testing and prescription.

[14] Fontes MT, Silva TL, Mota MM, Barreto AS, Rossoni LV, Santos MR (2014). Resistance exercise acutely enhances mesenteric artery
insulin-induced relaxation in healthy rats. Life Sci.

[15] Barauna VG, Batista ML, Costa Rosa LF, Casarini DE, Krieger JE, Oliveira EM (2005). Cardiovascular adaptations in rats submitted to a
resistance-training model. Clin Exp Pharmacol Physiol.

[16] Menezes IA, Moreira IJ, Carvalho AA, Antoniolli AR, Santos MR (2007). Cardiovascular effects of the aqueous extract from Caesalpinia
ferrea: involvement of ATP-sensitive potassium channels. Vascul Pharmacol.

[17] Furchgott RF, Zawadzki JV (1980). The obligatory role of endothelial cells in the relaxation of
arterial smooth muscle by acetylcholine. Nature.

[18] Holécyová A, Török J, Bernátová I, Pechánová O (1996). Restriction of nitric oxide rather than elevated blood pressure
is responsible for alterations of vascular responses in nitric
oxide-deficient hypertension. Physiol Res.

[19] Kopincová J, Púzserová A, Bernátová I (2012). L-NAME in the cardiovascular system - nitric oxide synthase
activator?. Pharmacol Rep.

[20] dos Santos FM, Martins Dias DP, da Silva CA, Fazan R, Salgado HC (2010). Sympathetic activity is not increased in L-NAME hypertensive
rats. Am J Physiol Regul Integr Comp Physiol.

[21] Somlyo AP, Somlyo AV (1994). Signal transduction and regulation in smooth
muscle. Nature.

[22] Hong E, Larios F, Gómez-Viquez NL, Huang F, Bravo G (2011). Role of alpha adrenoceptors and nitric oxide on cardiovascular
responses in acute and chronic hypertension. J Physiol Biochem.

[23] Tabernero A, Giraldo J, Vila E (1996). Effect of NG-nitro-L-arginina-metil-ester (L-NAME) on functional
and biochemical alpha 1-adrenoceptor-mediated responses in rat blood
vessels. Br J Pharmacol.

[24] Heijenbrok FJ, Mathy MJ, Pfaffendorf M, van Zwieten PA (2000). The influence of chronic inhibition of nitric oxide synthesis on
contractile and relaxant properties of rat carotid and mesenteric
arteries. Naunyn Schmiedebergs Arch Pharmacol.

[25] Chies AB, de Oliveira AM, Pereira FC, de Andrade CR, Corrêa FM (2004). Phenylephrine-induced vasoconstriction of the rat superior
mesenteric artery is decreased after repeated swimming. J Smooth Muscle Res.

[26] Braunstein TH, Inoue R, Cribbs L, Oike M, Ito Y, Holstein-Rathlou NH (2009). The role of L- and T-type calcium channels in local and remote
calcium responses in rat mesenteric terminal arterioles. J Vasc Res.

[27] Fellner SK, Arendshorst WJ (2010). Complex interactions of NO/cGMP/PKG systems on Ca2+ signaling in
afferent arteriolar vascular smooth muscle. Am J Physiol Heart Circ Physiol.

[28] Bank N, Aynedjian HS, Khan GA (1994). Mechanism of vasoconstriction induced by chronic inhibition of
nitric oxide in rats. Hypertension.

[29] Chen SJ, Wu CC, Yen MH (2001). Exercise training activates large-conductance calcium-activated
K(+) channels and enhances nitric oxide production in rat mesenteric artery
and thoracic aorta. J Biomed Sci.

[30] Delp MD, McAllister RM, Laughlin MH (1993). Exercise training alters endothelium-dependent vasoreactivity of
rat abdominal aorta. J Appl Physiol (1985).

[31] Fagard RH, Cornelissen VA (2007). Effect of exercise on blood pressure control in hypertensive
patients. Eur J Cardiovasc Prev Rehabil.

[32] Mota MM, da Silva TL, Fontes MT, Barreto AS, Araújo JE, de Oliveira AC (2014). Resistance exercise restores endothelial function and reduces
blood pressure in type 1 diabetic rats. Arq Bras Cardiol.

[33] Miyachi M (2013). Effects of resistance training on arterial stiffness: a
meta-analysis. Br J Sports Med.

